# Advances in Zebrafish for Diabetes Mellitus with Wound Model

**DOI:** 10.3390/bioengineering10030330

**Published:** 2023-03-06

**Authors:** Bangchang Lin, Jiahui Ma, Yimeng Fang, Pengyu Lei, Lei Wang, Linkai Qu, Wei Wu, Libo Jin, Da Sun

**Affiliations:** 1 Sir Run Run Shaw Hospital, Zhejiang University, Hangzhou 310000, China; 2 Institute of Life Sciences & Biomedical Collaborative Innovation Center of Zhejiang Province, Wenzhou University, Wenzhou 325035, China; 3 Key Laboratory for Biorheological Science and Technology of Ministry of Education, State and Local Joint Engineering Laboratory for Vascular Implants, Bioengineering College of Chongqing University, Chongqing 400044, China; 4 Wenzhou City and WenZhouOuTai Medical Laboratory Co., Ltd. Joint Doctoral Innovation Station, Wenzhou Association for Science and Technology, Wenzhou 325000, China

**Keywords:** adult zebrafish, zebrafish larvae, diabetic wound, delayed wound healing, model animal

## Abstract

Diabetic foot ulcers cause great suffering and are costly for the healthcare system. Normal wound healing involves hemostasis, inflammation, proliferation, and remodeling. However, the negative factors associated with diabetes, such as bacterial biofilms, persistent inflammation, impaired angiogenesis, inhibited cell proliferation, and pathological scarring, greatly interfere with the smooth progress of the entire healing process. It is this impaired wound healing that leads to diabetic foot ulcers and even amputations. Therefore, drug screening is challenging due to the complexity of damaged healing mechanisms. The establishment of a scientific and reasonable animal experimental model contributes significantly to the in-depth research of diabetic wound pathology, prevention, diagnosis, and treatment. In addition to the low cost and transparency of the embryo (for imaging transgene applications), zebrafish have a discrete wound healing process for the separate study of each stage, resulting in their potential as the ideal model animal for diabetic wound healing in the future. In this review, we examine the reasons behind the delayed healing of diabetic wounds, systematically review various studies using zebrafish as a diabetic wound model by different induction methods, as well as summarize the challenges and improvement strategies which provide references for establishing a more reasonable diabetic wound zebrafish model.

## 1. Introduction

Diabetes is a debilitating disease that has a significant economic impact on global health systems [1]. According to the International Diabetes Federation, 463 million adults had diabetes in 2019 and this number is expected to reach 700 million by 2045 [2,3]. The healing of a normal wound generally happens in four stages: hemostasis, inflammation, proliferation, and remodeling [4]. However, diabetic hyperglycemia leads to various systemic complications, resulting in a series of local lesions in the wound microenvironment, including chronic inflammation, angiogenesis, hypoxia-induced oxidative stress, neuropathy, impaired signal transduction of advanced glycation end products (AGEs) and neuropeptides. Eventually, diabetic wounds are characterized by impaired healing, prolonged inflammation, and reduced epithelialization dynamics [5,6,7,8,9,10]. In addition, even when blood glucose control is achieved medically, diabetes complications progress unhindered through metabolic memory phenomena, including delayed wound healing [11]. In accordance with N. Graves et al. it was epidemiologically concluded that more than 80% of amputations caused by diabetic foot ulcers (DFUs), often need to extend the time of hospitalization which aggravates the amputation patients’ economic burden. The global prevalence of DFU has reached 6.3%, about 50% of people in the event of a DFU within five years of death [12,13]. A better understanding of the pathological processes and mechanisms contributing to ulcers in diabetic patients and the influence on wound healing is therefore urgently needed to improve treatment strategies. Based on the available studies, the known mechanisms of delayed diabetic wound healing are listed in Table 1.

**Figure 1:**
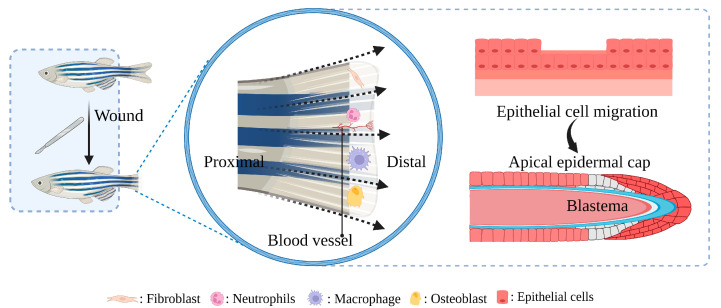
The role of participating in a zebrafish caudal fin regenerative wound healing model. Within 1–3 h-post-amputation (hpa), epithelial cells migrate to cover and close the wound. By 18–24 hpa, an apical epidermal cap is formed, and a mass of undifferentiated mesenchymal cells called the blastema accumulates underneath the apical epidermal cap. At 24 hpa the blastema cells segregate into two morphologically indistinct compartments: a slowly proliferating distal blastema and a rapidly proliferating proximal blastema. After 48 hpa the regeneration program is installed and the regenerative outgrowth continues until the original tissue architecture is reconstituted.

For preclinical studies of diabetic wound therapy, appropriate animal models are essential; after all, studies in vitro cannot fully capture the complexity of the diabetic wound environment [28]. Mice and rats have loose skin and high hair density. Pigs have high cost, thick dermis, and slow passage speed. Rabbit genetic susceptibility is limited. Monkey ethics are very demanding. Guinea pigs are generally limited to the therapeutic effects of vitamin C [29,30,31,32]. In recent years, zebrafish (*Danio rerio*) have become increasingly popular as animal models in biomedical, toxicological, and pharmacological research [33,34], much of which has to do with the highly conserved nature of pathways and genes, good genetic susceptibility, low cost, and rapid passage [35,36,37,38]. As opposed to other wound healing models listed above, zebrafish and mammals utilize a very similar principle to close their epidermis; their caudal fin wounds re-epithelialize rapidly, making them an ideal model for studying wound healing—the healing process of caudal fin injury is shown in Figure 1. The discrete phases of the healing process for separate research, as well as the development of gene editing technology (such as CRISPR-Cas9), allow for the understanding of the mechanism at great convenience and accuracy [39]. Further, researchers are increasingly paying attention to wound healing in zebrafish due to the construction of zebrafish diabetes models that are rapidly progressing [40,41]. This review introduces the specific mechanism of diabetic wound healing delay and zebrafish model in the study of diabetic wounds, showing the advantage of, and systematically summing up the model in different stages of the development of the diabetic wound application progress. The challenges and potential improvement strategies, and the future trend of the model are also discussed.

## 2. Mechanism of Delayed Healing of Diabetic Wounds

Usually, the healing process begins with hemostasis to prevent blood loss and microbial invasion of the wound. This phase follows and overlaps with the inflammatory stage, in which proinflammatory neutrophils are up-regulated initially, and then macrophages clean up fragments and pathogens as well as growth factors, other cytokines, and cells. The proliferative phase overlaps with the inflammatory phase, during which new tissues, new blood vessels (angiogenesis), and matrix structures are activated to fill the wound [42,43]. The final remodeling stage increases the tensile strength of the extracellular matrix (ECM) and reduces the blood supply to the damaged area [21,44,45].

**Figure 2:**
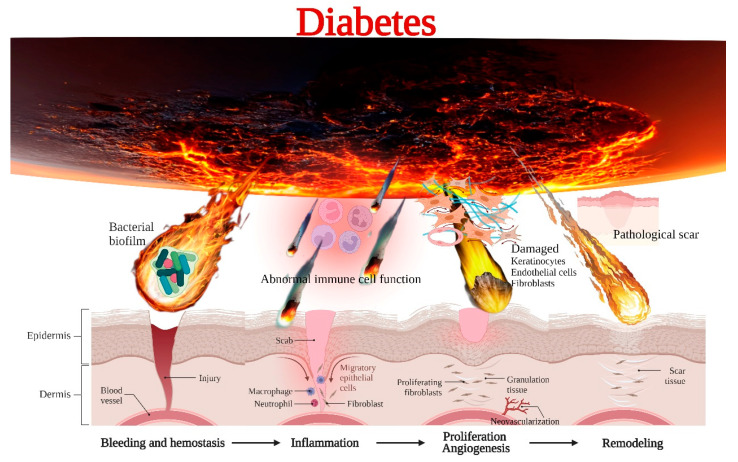
The molecular and cellular mechanisms in delayed diabetic skin wound healing.

Diabetes is the most common heterogeneous metabolic disorder, which is associated with disorders of glucose, lipid, and protein metabolism [46], characterized by elevated blood glucose or insulin response to tissue, which can lead to many complications, including diabetic skin wounds or ulcers [47]. Diabetic skin ulcers are characterized by painful ulcers with disintegration of dermis (including epidermis, dermis and, in many cases, subcutaneous tissue) [48]. At present, the research on the pathology of DFU is mainly focused on immune dysfunction, microbial invasion, impaired cell proliferation, angiogenesis, and pathological scar [49,50,51,52,53]. The molecular and cellular mechanisms in delayed diabetic skin wound healing are shown in Figure 2.

### 2.1. Immune Dysfunction

The inflammatory phase of the wound can last for weeks or even months under the influence of diabetes [54,55]. The repair process (bacterial defense, cell proliferation, and collagen synthesis) requires energy, and the strictly oxygen-dependent NADPH-linked oxygenase will produce ROS during this period. Many pathways (polyol pathway, hexosamine pathway, etc.) related to oxidative stress of AGEs are maladjusted, resulting in NADPH depletion, decreased glutathione production, activation of protein kinase C and NADPH oxidases, and excessive production of oxygen free radicals and ROS, which further magnify the inflammatory response under high glucose conditions [7,56]. The functional impairment of neutrophils and macrophages was manifested in the continuous activation of citrulline histone H3, NETosis, and irregular release of NETs in diabetic wounds. Macrophages were affected by increased secretion of prostaglandin E2/D2 and excessive accumulation of AGEs, resulting in weakening of burial and phagocytosis of polymorphonuclear leukocytes such as apoptotic neutrophils, which remained in the M1 phenotype for a long time [17,21,57]. Moreover, the low number of primordial T cells and the poor range of T cell receptor (TcR-V β) in diabetic patients lead to the accumulation of effector T cells, which, together with high levels of AGEs, result in the increase of proinflammatory cytokines such as IL-6 and TNF-α, which cause the interruption of the inflammatory cascade, excessive inflammation, and insulin resistance. There are also other disorders of pro-inflammatory factors, such as abnormal expression of macrophage inflammatory protein (MIP)1α, MIP2, CX3C chemokine ligand 1, DNA methyltransferases (DNMTs), IL-12, NLRP3 [58], reduced level sof human β-defensin 1, 2 and 3 and Setdb2, irregular activation of signal transducer and activator of transcription 3 (STAT3), and decline in the activation of AKT/serine/threonine protein kinase B and NF-κB [21,22]. All these collectively contribute to interruption of healing in diabetic wounds. The RNA expression of macrophage migration inhibitory factor (MIF) gene is decreased in diabetic patients, and the decrease of MIF level may be the reason for the damage of the endothelial progenitor cell production and the healing process [21,59,60]. Hyperglycemia and oxidative stress can also cause abnormal glycosylation of nerve cell proteins and abnormal activation of protein kinase C, leading to neurological dysfunction and ischemia, while peripheral neuropathy leads to weakening of motor and foot sensory structures. This increases the risk of ulcers caused by repeated mechanical stress [59].

### 2.2. Microbial Invasion

The ability of diabetic patients to stimulate immune response is limited, and toll-like receptors (TLRs) are down-regulated in diabetic wounds, which can damage the innate immune system and inflammatory response, decrease the number of CD4+ T cells, and reduce chemotaxis. This delays the recruitment and immune response of various inflammatory cells, resulting in bacterial susceptibility, bacterial connection, and biofilm formation in the wound. These biofilms protect microorganisms from antimicrobial agents and immune systems, and disrupt the healing process [15,21]. The minimum inhibitory concentration and minimum bactericidal concentration of bacteria in biofilm may be as high as 10–1000 times compared with planktonic bacteria [61]. The pH of the wound becomes alkaline under persistent infection, inhibiting physiological processes such as angiogenesis, epithelial hyperplasia, oxygen release, and bacteriostasis [14]. It is the most common cause of lower limb amputation in diabetic wounds.

### 2.3. Impaired Cell Proliferation and Angiogenesis

In the proliferative stage, the cascade mediated by different matrix metalloproteinases, cytokines, inflammatory cells, keratinocytes, fibroblasts, and endothelial cells is the basis of successful wound healing [62,63,64,65]. On the one hand, keratinocyte and fibroblast proliferation and migration provide basic conditions for ECM and re-epithelialization [66,67,68]. On the other hand, a continuous supply of blood provides adequate oxygen and cytokines, which are necessary for tissue regeneration. Wound areas with active proliferating fibroblasts can only be seen when pO_2_ is above 15 mm Hg [7,23]. Further, keratinocyte, fibroblast proliferation and migration provide basic conditions for ECM and re-epithelialization of ECM [69]. Decreased levels of heat shock proteins (HSPs) (HSP90, HSP70, HSP47, and HSP27) and their downstream molecules TLR4 and p38-mitogen-activated protein kinases affect procollagen synthesis and protein homeostasis [21,70]. Irregular pro-inflammatory response can activate the irrational upregulation of activating transcription factor-3 and inducible nitric oxide synthase (iNOS), accompanied by the increasing level of free radicals and the upgradulating activities of caspase-3, -8 and -9. High oxidative stress results in the down-regulation of nuclear factor E2-related factor 2 (affecting the expression of MMP-9, transforming growth factor-beta (TGF-β), migration and proliferation-related genes). Impaired cell differentiation and remodeling are associated with abnormal Bcl2, keratin K16, notch junction protein 43, and platelet reactive protein- 1 expression. In addition, the expression of angiopoietin-like 4, one of the stromal cell proteins, is difficult to up-regulate in diabetic wounds as occurs in normal wounds. The activation of the JAK1/STAT3/iNOS signaling pathway is blocked, and the production of NO is inhibited, hinting to angiogenesis and re-epithelialization [21,23]. High glucose concentration change the level and the expression of MMPs, directly through the activation of inflammatory cells, induce sustained high levels of proinflammatory cytokines, promote fibrosis, and reduce the influence of the expression of TIMPs, as well as through the formation of the advanced glycosylation product indirect influence of MMPs. A study stated that metalloproteinases levels in chronic wound fluid are almost 60 times that of an acute wound. This substantially eliminates growth factors, receptors, and matrix proteins at the wound site [23].

### 2.4. Pathological Scar

The remodeling phase requires the synthesis of new collagen and concurrent collagen degradation, a process mediated by MMPs. Type III collagen is gradually replaced by type I collagen with greater tensile strength. The presence of myofibroblasts also causes wound contraction, contributing to potential scar formation [23]. However, premature senescence of fibroblasts occurs under high glucose conditions, which is associated with persistent oxidative stress and inflammation [42]. At the same time, pathological skin scarring exhibits excessive accumulation of fibroblasts and ECM (mainly type I collagen). The vertical growth of pathological scars generally subsides after a few years; however, the joint site can lead to contracture, severely limiting function, requiring surgical treatment, and seriously affecting appearance and normal life [42]. Several lines of evidence suggest that inflammatory bodies in local fibroblasts are involved in skin fibrosis by inducing these normally stationary cells to differentiate into pathogenic myofibroblasts, resulting in high levels of ECM. This inflammasome activation in these non-immune cells triggers skin fibrosis, and subsequent inflammasome activation in immune cells amplifies the local inflammatory response [58].

The pancreas of zebrafish and some insulin-sensitive peripheral tissues, such as liver and muscle, are evolutionally conservative. Some important genes related to sugar metabolisms, such as hexokinase and glucose transporter genes, are actively expressed in zebrafish, and the deletion of these genes causes a series of severe neurological defects in zebrafish embryos. In addition, some of the key mechanisms involved in the regulation of sugar metabolism are very similar to those in other mammals, and studies have shown that NF-κB-inducing kinase level in juvenile zebrafish was induced by drugs, while the NF-κB bypass signaling pathway was exogenously activated. The blood glucose level of juvenile zebrafish was significantly increased, and β-cell function was abnormal after 6 h. Although intracellular insulin production was normal, its secretion was obviously blocked [11,71,72,73,74]. Therefore, zebrafish as a model animal object for the exploration of diabetic wound mechanisms and drug screening will likely be the mainstream direction in the future.

## 3. The Advantages of Zebrafish for Diabetic Wound Healing

**Figure 3:**
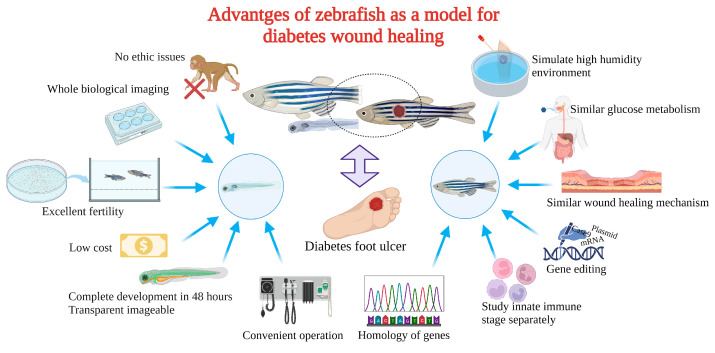
Advantages of zebrafish for diabetes mellitus with wound model.

Zebrafish as a model for studying diabetic wound healing has many unique advantages (Figure 3): Zebrafish larvae are only subject to animal experimentation regulations upon reaching 120 h post fertilization and starting to live free, as stipulated by the directive of the European Union on the Protection of Animals used for scientific purposes. As the volume is small, whole-organism imaging can be performed in a multiwell plate; the cost is low; the reproduction ability is strong (about 200 eggs per female per week) [75]; the generation time is short, generally 3–4 months. Thus, selection experiments can be conducted; larvae developed rapidly, and fully developed juveniles can be obtained 48 h after hatching. The embryonic and juvenile stages are transparent throughout the body, making real-time imaging of target organs possible, giving ease of embryonic manipulation, and the possibility of screening therapeutic agents at a low cost [76,77,78]. All of the above contribute to their increasing popularity for high-throughput and high-content assays. On the other hand, as a result of the zebrafish genome sequence, 71% of human proteins and 82% of disease-causing human proteins have been found to be orthologous in zebrafish, with significant homology to human proteins. The mechanisms of infection and inflammation caused by innate immune responses can be isolated and studied in the early stages of development without the complications associated with adaptive immune responses. It is suitable for the study of gene expression regulation by gene specific knockout technique or by mRNA or plasmid overexpression of protein. There is good genetic control and in the embryonic and larval stages, the skin of zebrafish is already composed of a superficial peritrind, a middle epidermal double layer, and a basal layer attached to the basal membrane, while its multilayer epidermis is formed during the metamorphosis on the 25th day after fertilization. At the same time, fibroblasts penetrate the dermis, take over the collagen produced by keratinocytes in the basal layer, form locally thickened dermal papillae, and begin to scale. In short, the skin structure of zebrafish is very similar to that of human beings, and the basic principle of the wound healing mechanism is conservative between human and zebrafish: there are discrete stages of healing, allowing specific processes to be studied separately; a remarkable ability to regenerate new fin tissue after amputation is retained, and its caudal fin has a relatively simple but symmetrical structure, including epidermis, blood vessels, nerves, pigment cells, and fibroblasts [39,79,80,81,82,83]. Since genetic engineering technology has developed rapidly, especially in CRISPR-Case9 (efficient multiple gene targeting, zebrafish mutant strain breeding, etc.), it has been possible to produce all kinds of simple alternative cell spontaneous fluorescence zebrafish varieties (with fluorescent markers of neutrophils and macrophages of transgenic zebrafish, and the development of reporter cell lines that tag specific tissues, including epithelial tissues) [84,85,86,87], as well as other manipulation techniques, such as mRNA injection and morpholine injection [88], and their intact immune systems. Moreover, it is convenient to obtain specific mutant zebrafish such as the rag1^−/−^ mutant zebrafish, which is the only animal model that can be used for the study of T-cell and B-cell immune response defects [40]; The glucose metabolism of adult zebrafish and its development during embryonic development are closely related to that of humans and other mammals. Under physiological conditions, the blood glucose level of zebrafish is approximately 60 mg/dL (3.3 mmol/L), which is dynamically regulated by feeding and fasting. The model was also extended to include factors associated with patients with diabetes mellitus or other metabolic disorders such as cholesterol and triglycerides, body weight, body mass index (BMI), and lean body mass; in zebrafish, it took only 2 days to induce hyperglycemia after injection of pdx1 morpholine. It took weeks for scientists to observe changes in the kidneys caused by hyperglycemia in mice and rats. Zebrafish, however, showed kidney changes within two days. Since diabetic zebrafish cells can easily be transplanted into healthy zebrafish for subsequent tracking and analysis, zebrafish are also excellent models to study glucose memory effects. Meanwhile, in the high fat diet (HFD)-induced type 2 diabetes model, zebrafish demonstrated HFD-induced changes in a much shorter period of time, such as liver changes after two months. Furthermore, when experimental hyperglycemia was determined by streptozotocin injection, a distinct impairment of caudal fin regeneration was observed [89]. It is worth mentioning that pressure sores caused by long-term bed rest are often associated with the hospitalization of diabetic patients for foot ulcers. These pressure sores are closely related to moisture and are more difficult to heal under the influence of diabetes [90,91]. The zebrafish wound model has always maintained a high humidity environment, which is favorable for the degree of reduction of the diabetic pressure sores model.

## 4. Construction and Application of Zebrafish Diabetic Wound Model

**Figure 4:**
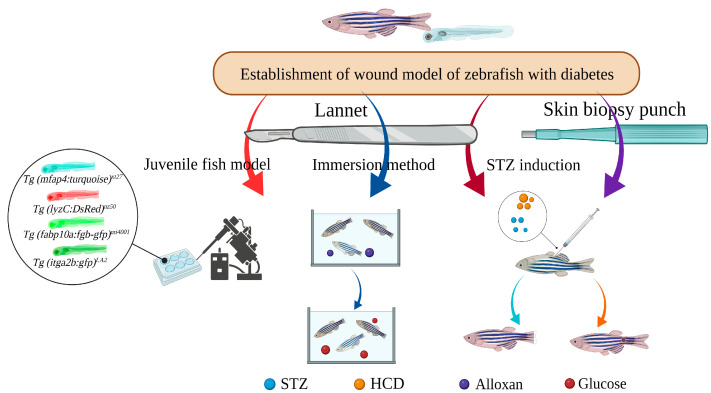
Application of zebrafish and their larvae in diabetic wounds.

Zebrafish have been applied as ideal model animals for diabetic wound studies. These construction and application are described in Figure 4.

### 4.1. STZ-Induced Caudal Fin Regeneration Model of Zebrafish with Type 1 Diabetes

Zebrafish respond efficiently and rapidly to streptozotocin (STZ) injection induced diabetes, and hypercholesterolemia caused by high cholesterol diet (HCD)—associated with a high risk of DFUs) Cho et al. [92] injected 30 μL 5 mM citrate buffer containing 0.3% STZ into the subcutaneous tissue adjacent to the abdomen for eight consecutive days utilizing a 26-needle micro-syringe. Prior to that, they were given a 4% cholesterol high cholesterol diet (HCD) for 4 weeks, consisting of normal diet (ND) alone group, ND+STZ group, HCD alone group. and HCD+STZ group. Finally, patients were treated with Heberprot-P75^®^, Easyef^®^ (two commercial epidermal growth factor (EGF) products, intraperitoneal injection of 10 μL, 50 μg/mL) and PBS on day 3, 5 and 7, respectively. As a result of treatment with PBS in the ND+STZ and HCD+STZ groups, adult fish showed serious delays in healing, as well as multiple cracks which is the typical damage pattern induced by STZ injection in diabetic zebrafish on their caudal fins. However, no cracks appeared in the HCD alone group [93]. Heberprot-P75^®^ showed caudal fin regeneration activity 2.1 times higher than Easyef^®^ (ND+STZ group) and 1.7 and 1.5 times higher than the Easyef^®^ group and PBS group (HCD+STZ group) under the same injection and amputation regimen, with more distinct and clean regeneration modes [93]. Intine et al. injected 0.35 mg/g of STZ intraperitoneally for 1, 3, and 5 days and maintained the injection weekly with the tank temperature maintained at 22–24 °C, as well as amputating the caudal fin in a straight line using a sterile size 10 scalpel, proximal to the first lepidotrichia branching point to obtain an adult zebrafish wound model of type I diabetes bearing an average blood glucose of more than 300 mg/dL, impaired caudal fin regeneration, accumulation of AGEs, and epigenetic changes including genome-wide demethylation. At 21 days to stop the injection of STZ, and restore normal blood insulin and glucose control through pancreas regeneration, and obtain the metabolic memory (MM) fish, whose limb regeneration was still the same as the state of acute diabetes damage, even at 30, 60, 90 days, and this affects the genetic to daughter cells [11], as well as bnormal DNA methylation was also retained, but AGEs did not accumulate and ROS induced stress signals did not increase. In conclusion, restoring physiologically normal glycemic control may not save altered target tissue from diabetes-induced changes [94]. Microarray transcript analysis was also performed applying this model and revealed abnormal expression of the following genes in T_0_: (1) developmental transcription factors (such as *SOX3*, *emx2*, *dlx4a*, etc.), (2) DNA modification related genes (*DNMT1*, *apex1*, *MCM2*, *uhrf1*, etc.), and (3) stress response/trauma repair genes (*HSP70*, *hspd1*, *MMP13*, etc.). Some genes in the earliest stage of fin regeneration (T_12_) overlap with genes that are abnormally expressed at the T_0_ time point (*ets1a*, *mmp13*, and *inhbaa*), while there are others, namely: (1) development-related transcription factors such as homeobox genes, (2) signal transduction molecules such as inhibin (*inhbaa*), EGF-like protein (*hbegf*) and jag1b of notch pathway, and (3) ECM-related genes, For example, at *MMP13*, *vcanb* and *chst11*, WNT ligand (*wnt2*) and a canonical wnt receptor (*fzd9*) are also found to change at the T_24_ time point. A connective tissue growth factor (*ctgf*) was down-regulated (−1.8 in diabetes mellitus (DM) and –1.7 in MM) and *FGF10a* was up-regulated. At T_48_, BMP inhibitor bambi and the notch inhibitor lunatic fringe (*lfng*) were up-regulated (8.6 fold in DM and 1.7 fold in MM). The range of gene expression change in the DM state was almost always significantly greater than that in the MM state—mean multiples change of up-regulated genes: 7.0 (DM): 2.0 (MM), mean multiples change of down-regulated genes: 2.4 (DM): 1.9 (MM)—but altered expression in the MM state was still sufficient to alter normal signaling pathways. In addition, CpG islands were identified for 61 of the 71 genes with abnormal expression associated with DNA methylation, and seven of them were found to have at least a two-fold reduction in methylation status within their promoter region. In the follow-up study of Sarras et al., the *Tg* (*fli1a:nEGPF*) zebrafish line20 was used to monitor in vivo angiogenesis. The wound angiogenesis induced by hyperglycemia was impaired in adult zebrafish, and this damage continued even after the fish returned to normal blood glucose state. Inhibition of poly (ADP-ribose) polymerase (PARP) activity can effectively prevent demethylation events and prevent angiogenesis defects [94,95]. Thus, this model has the following attributes: avoids the complex peaks and troughs of glycemic control that might occur in animals requiring exogenous insulin in terms of examining metabolic memory; eliminates background stimuli of the prior diabetic state and examines the pure epigenetic factors of metabolic memory; only takes about 80 days from diabetes induction to metabolic memory examination; allows easy genetic and experimental manipulation; allows for future drug discovery [11,71]. At the same time, it was shown that high blood glucose did not seem to result in an increase in cell death, but reduced the proliferative ability of regenerated limbs with a similar treatment [96]. Another study applying transgenic zebrafish *Tg (fli1:EGFP)* demonstrated an increase in the percentage of GFP-positive endothelial cells accumulated at the S and G2/M stages after treatment of simplified 2-herb formula (NF3) embryos, a significant increase in the mRNA of *VEGF*, *VEGFR2* (*Flk-1*), *fgf1 and bRaf* expression levels, and an upregulation trend of *VEGFR1 (Flt-1)*, *fgfr2*, *cpl2* and *mknk2b* expression, which provides a scientific basis to support NF3 as a potential therapy for the treatment of DFU [97].

### 4.2. Caudal Fin Model of Type II Diabetes in Adult Zebrafish Induced by Alloxan and Glucose Combined with Aqueous Solution Exposure

Aquaporin (AQP) and GLUT1, both present in the gill and skin epithelium, are thought to be responsible for the production of HG zebrafish following the application of alloxan and glucose in water. Wibowo et al. combined 0.4% Alloxan and glucose (E-Merck) % solution, and placed adult zebrafish aged from 3 to 6 months in 100 mL Alloxan solution for 1 h a day for 5 days, and then transferred 2L 2% glucose solution for 24 h for 6 days, as well as amputating using a lancet (Aesculap Scalpel Handle No. 3 and Aesculap Scalpel Blade No. 10 of B BRAUN) at the first or the second segment below the level of the first ray bifurcation. The expressions of *shha*, *igf2a*, *bmp2b,* and *col1a2* were down-regulated in the experimental group, which may be related to the glucose metabolism inducing the generation of superoxide or ROS, the inhibition of GAPDH, the accumulation of methyl glyoxal, and the disruption of hypoxia inducible factor-1α transcription factor stability, leading to the transcriptional inhibition of some of the above target genes. The percentage of caudal fin regeneration and the expression of *shha*, *igf2a*, *bmp2b,* and *col1a2* were increased after treatment with 15 ppm propolis ethanol extract [98].

### 4.3. Caudal Fin Regeneration Model of Zebrafish Juvenile Type II Diabetes Induced by Single Immersion or Injection of Glucose

With zebrafish, each process can be studied separately, which allows us to observe wound healing more directly. Morris et al. studied the wound model of hyperglycemic transgenic *Tg (mfap4:turquoise)^xt27^* induced by immersion (5% glucose) and injection (15 nmol). Macrophages of transgenic *Tg (mfap4:turquoise)^xt27^* juveniles were labeled with turquoise fluorescent protein, neutrophils of *Tg (lyzC:DsRed)^nz50^* juveniles were labeled with DsRed fluorescent protein, and reduced neutrophils were found in the juveniles injected with glucose. That is, innate immune cell development is affected. *Tg (itga2b:gfp)^LA2^* juvenile tail fin transection was observed to stop bleeding, while injection and immersion showed reduced platelet accumulation. At the same time, the accumulation of fibrin was reduced by using *Tg (fabp10a:fgb-gfp)^mi4001^*, which expresses fluorescently tagged fibrinogen and allows visualization of clots. Finally, the infusion of glucose at 0 day post fertilization (dpf) and the feeding of a HFD at 5 dpf found a significant acceleration of lipid accumulation after only one day of feeding, providing a more rapid model for studying lipid accumulation [99].

Besides the above-mentioned diabetes peripheral neuropathy caused by the adverse effects of wound healing, there was the application of the zebrafish larvae percutaneous absorption ability to extend the study of high blood glucose. D-glucose treatment for five days in 60 mM, axon desquamation, neural glial sheath surrounding obstacles, sports axon myelin formation decreases, sensory neuron localization error [100], that provide a new research model for in-depth understanding of peripheral nerve structural changes following induction of hyperglycemia and for applying therapeutic measures to provide joint solutions for the healing of diabetic wounds.

### 4.4. Skin Wound Model of Adult Zebrafish Type I Diabetes Induced by STZ Injection

The full-layer wound healing mechanism of zebrafish is very similar to that of humans. On the one hand, in the embryonic and larval stages, the skin of zebrafish is already composed of the outer layer of peritrind, the middle layer of epidermis, and the basal layer attached to the basal membrane. In the process of metamorphosis on the 25th day after fertilization, multiple layers of epidermis are formed. At the same time, fibroblasts penetrate the dermis and take over collagen produced by basal keratinocytes to form locally thickened dermal papilla and scale, which is very similar to human skin structure. On the other hand, skin healing of zebrafish involves activation of signal transduction pathways downstream of hydrogen peroxide, including epidermal growth factor EGF, forkhead box-1, and IkappaB kinase-alpha. EGF regulates TGF-β through ERK1/2 and EGFR signal transduction. Damaged cells bind to EGFR to trigger cascades (such as TGF-β/integrin and ROCK/JNK pathways) to induce DNA synthesis and cell proliferation at the wound site. In addition, Wnt/β-catenin is upregulated to promote healing. These pathways all overlap with human wound-healing mechanisms [83], so the research object of wound healing based on zebrafish skin is increasingly extensive. Laser has been used to form a single wound on the side of zebrafish for drug screening, and the method of soaking and exposing to vibrio parahaemolyticus and mycobacterium marine infection on the back after skin bruising has been used to establish a chronic wound model to study the healing mechanism [101,102,103]. At the same time, combined with the characteristics of delayed wound healing in zebrafish after diabetes similar to that in humans [83], the zebrafish skin diabetic wound model will be a promising research model in the future. A circular wound with a diameter of 1 mm was created on the posterior lateral side of the body wall of the proximal tail fin of zebrafish with induced diabetes by a skin biopsy needle. Comparing the remaining open wound area (24 h after injury) with the original wound area, it was proved that the wound healing speed of DM zebrafish was slower than that of normal zebrafish. This reduction remained the same in 60-day MM fish. These data clearly indicate that skin wound healing remains impaired and affected by MM after glycemic homeostasis is achieved [71]. In the field of diabetic wounds, however, the application of the zebrafish skin model is still in its infancy. Future studies focusing on this modeling method will provide a great basis and convenience for the mechanism exploration of diabetic wounds and drug screening. In conclusion, the zebrafish diabetic wound model has been widely applied, and it will be very promising to conduct in-depth research on pathological mechanisms and drug screening and evaluation through this model in the future.

## 5. Challenge and Improving Strategies

**Figure 5:**
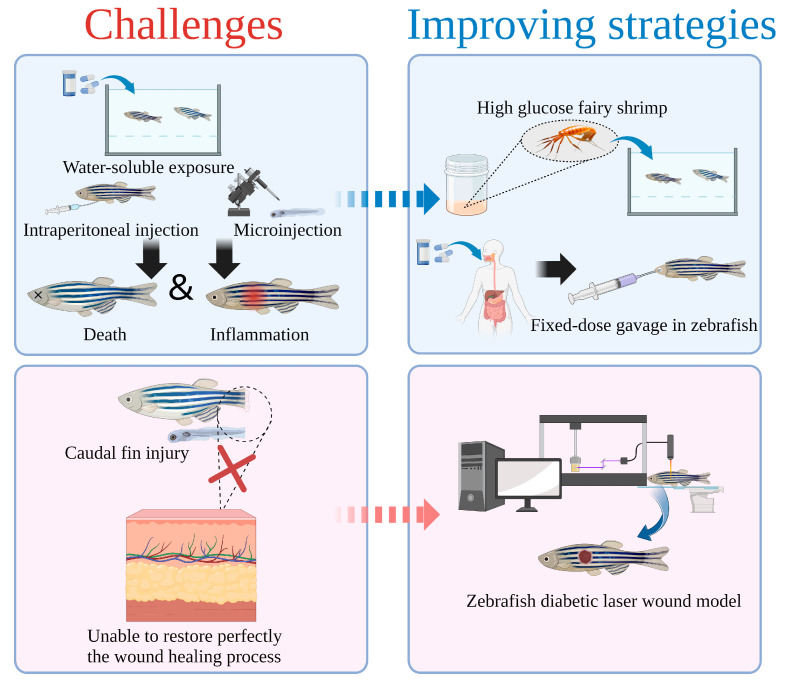
The challenge and improvement strategies of zebrafish as a model for diabetic wound healing.

Diabetic zebrafish are currently constructed primarily through water-soluble exposure and injection (Figure 5). Modeling with this method may lead to irreversible damage, abnormal swimming and gill function, or even death for zebrafish because of their limited tolerance [104,105,106]. Wang Lei et al. incubated 20 g fairy shrimp eggs in a 2 L incubator (1.5 L DDH_2_O, 8 g salt for incubation), and added 500 mL 80% glucose 16 h later to ensure a final concentration of 20%, as well as collecting the fairy shrimp after 24 h of incubation, rinsed them twice with DDH_2_O, immediately placed them in liquid nitrogen, and then freeze-dried them for 36–48 h. Finally, high-glucose fairy shrimp were obtained. The high glucose fairy shrimp were fed 40 g/kg every day, and a diabetic zebrafish model with 100% survival rate was established [107]. As well, despite the convenience of water-soluble exposure, glucose and drugs will not be effectively absorbed quantitatively. In addition, the physical and physiological characteristics of the drugs themselves may also adversely affect the skin of zebrafish, causing inflammatory damage, for instance [108]. Thus, zebrafish gavage is considered an effective method in the future for quantifying experimental doses of zebrafish [109].

At the same time, current zebrafish diabetic wound research focuses on caudal fin regeneration. This method, however, is difficult for observation of the reconstructed structure (epidermis and dermis) in a more detailed way under the microscopic field of view, which is unfavorable to the restoration of the human skin repair process. Much literature has been reported through the application of laser full-thickness wounds that can be quickly and reproducibly introduced on the flank of adult zebrafish [39,110,111]. In addition to simulating wound healing to the greatest extent possible, this model is a great choice for studying diabetic wounds in zebrafish, since it could be utilized not only to study wound healing, but also evaluate fibroblast and keratinocyte growth and ECM reconstruction utilizing H&E staining and other methods [103]. Importantly, the size, diameter, and depth of the wound established by mechanical equipment can be carefully controlled, which is extremely advantageous for the study of screening specific drugs with different degrees of injury [39].

## 6. Conclusions

Delayed wound healing induced by diabetes results in a heavy burden on patients physically, mentally, and economically. To discover specific drugs with excellent efficacy, it is essential to investigate the specific mechanism behind the delayed feature utilizing appropriate preclinical research models. Zebrafish are characterized by high throughput, small ethical disputes, larval body transparency, quick development, fast and efficient establishment of a model of diabetes, convenient caudal fin regeneration of wound repair evaluation, and are the height of human gene homology, etc. In addition, quantitative gavage and feeding shrimp with high glucose can solve restrictions such as water-soluble exposure and potential damage by injection. Zebrafish will become an ideal model for diabetic wound model organisms in the future, bringing a new dawn for the screening of rapid healing treatment for diabetic wounds and the exploration of the underlying mechanism.

## Figures and Tables

**Table 1:** Factors and mechanisms of action causing delayed healing of diabetic wounds.

Inducement	Occurrence Stage	Mechanism	References
Persistent bacterial infection and biofilm	The whole process	Low CD4+ T cell counts;	[6]
		insufficient blood perfusion;	[14]
		abnormal pH (slightly alkaline);	[15]
		Abnormal function of mast cells.	[16]
Massive infiltration of neutrophils	Inflammation	Excessive neutrophil extracellular traps (NETs);	[7]
		Weak scavenging effect of macrophages;	[17]
		high pro-inflammatory factors levels; reactive oxygen species (ROS) vicious circle.	[18]
More pro-inflammatory macrophages and insufficient activity of anti-inflammatory macrophages	Inflammation	Impaired phagocytosis; decreased ability to polarize to anti-inflammatory state; excessive accumulation of AGEs.	[19,20,21,22]
Hyperkeratosis and insufficiency of wound margin; Stagnant reepithelialization	Proliferation	Weak migration ability of keratinocytes; Weak induction of regulated growth factors; Weak keratinocyte growth factors and fibroblast growth factors (FGFs) in wound; abnormal expression of miRNA.	[23], [24]
Defect of angiogenesis and local oxygen deficiency	Proliferation	Low expression of insulin-like growth factors-1 (IGF-1) and abnormal function of vascular endothelial cells;	[7]
		low vascular endothelial growth factor (VEGF) and VEGF receptor-2 (VEGFR-2) levels;	[25]
		fibrin restricts angiogenesis.	[26]
Delayed production of ECM and limited wound contraction	Proliferation and remodeling	Fibroblasts senescence; no response to growth factors such as FGF; less transformation to myofibroblasts.	[7], [27]
Delayed maturation of scar tissue	Remodeling	High level of matrix metalloproteinases (MMPs); low level of tissue inhibitor of matrix metalloproteinase-1 (TIMP-1); influence of microbial community.	[5], [7], [23]

## Data Availability

Not applicable.

## Abbreviations

AGEs	Advanced glycation endproducts
AQP	Aquaporin
BMI	Body mass index
DFUs	Diabetic foot ulcers
hpa	Hours-post-amputation
dpf	Day post fertilization
DM	Diabetes mellitus
DNMTs	DNA methyl transferases
EGF	Epidermal growth factor
ECM	Extracellular matrix
FGFs	Fibroblast growth factors
HSPs	Heat shock proteins
HCD	High cholesterol diet
HFD	High fat diet
iNOS	Inducible nitricoxidesynthase
IGF-1	Insulin-like growth factors-1
MIF	Macrophagemigration inhibitory factor
MIP	Macrophage inflammatory protein
MMPs	Matrix metallo proteinases
MM	Metabolic memory
NETs	Neutrophil extracellular traps
ND	Normal diet
PARP	Poly (ADP-ribose) polymerase
ROS	Reactive oxygen species
STAT3	Signal transducer and activator of transcription 3
STZ	Streptozotocin
TIMP-1	Tissue inhibitor of matrixmetallo proteinase-1
TLRs	Toll-like receptors
TGF-β	Transforming growth factor-beta
VEGF	Vascular endothelial growth factor
VEGFR-2	VEGF receptor-2
